# Outcome of Mini-Percutaneous Nephrolithotomy in Patients Under the Age of 18: An Experience With 112 Cases

**DOI:** 10.3389/fsurg.2021.613812

**Published:** 2021-06-15

**Authors:** Mohammad Mehdi Hosseini, Dariush Irani, Ala'a Altofeyli, Ali Eslahi, Mitra Basiratnia, Abdolreza Haghpanah, Ali Adib, Faisal Ahmed

**Affiliations:** ^1^Shiraz Nephrology-Urology Research Center, Shiraz University of Medical Sciences, Shiraz, Iran; ^2^Department of Urology, Shaheed Faghihi Hospital, Shiraz University of Medical Sciences, Shiraz, Iran; ^3^Shiraz Geriatric Research Center, Shiraz University of Medical Sciences, Shiraz, Iran; ^4^Student Research Committee, Shiraz University of Medical Sciences, Shiraz, Iran; ^5^Department of Urology, Urology Research Center, Al-Thora General Hospital, Ibb University of Medical Since, Ibb, Yemen

**Keywords:** minimal invasive, nephrolithiasis, nephrolithotomy, pediatrics, mini-percutaneous

## Abstract

**Purpose:** Renal calculi are becoming more common among children. Although, extracorporeal shock wave lithotripsy (ESWL) is the first choice in this age group, minimal invasive surgeries, such as percutaneous nephrolithotomy (PCNL), are indicated for some patients. Recently, PCNL devices have become smaller in size with acceptable efficacy and lower complications. We evaluated the outcomes and complications of mini-PCNL (MPCNL) surgery in our referral training centers.

**Materials and Methods:** Between September 2012 and January 2020, a total of 112 children under the age of 18, who had shown failure of ESWL, and/or their parents refused to do it, underwent MPCNL (15 Fr). The patients' profiles were reviewed for data collection including preoperative and stone data, operation information, and postoperative complications.

**Results:** Of 112 patients, 69 were boys, and 43 were girls. Their mean age was 8.6 years (14 months to 18 years). Mean stone size was 20 mm (14–34 mm). Seventy-four cases had renal pelvic stone, 22 had pelvis and lower pole, and 16 had staghorn. The mean operation time was 65 min (35–100 min), and mean radiation time was 0.6 min (0.2–1.4 min). Low-grade fever was detected in 14 patients (12.5%). Four patients needed blood transfusion and two had increased creatinine, which improved with conservative management. One patient developed urosepsis that resolved with antibiotic therapy. None of the patients had kidney perforation or other organ injury or death. Early stone-free rate (SFR) after operation was 90.2% (101 patients). Six patients had residual fragment <5 mm, which passed spontaneously in 2 weeks after operation (total SFR 95.3%). Three patients underwent second-look nephroscopy, and ureteroscopy was done for two patients due to migrated stone fragments to the distal ureter.

**Conclusion:** MPCNL is recommended as a safe alternative option for treatment of the nephrolithiasis in children with good outcome and acceptable complications.

## Introduction

Epidemiologic studies have demonstrated an increasing rate of pediatric urinary stone disease in the past decades (1–3). Although, pediatric urinary stone disease is less common in children than adults, it is more difficult to manage because of their urinary tract size and higher recurrence rate (4). The clinically insignificant stone fragments (<4–5 mm) that may be observed after treatment of adult stones can cause obstructive symptoms for pediatric patients and need for surgical intervention. However, spontaneous passage of a renal stone is more likely in children than in adults (5). Hence, the goals of surgical management of pediatric urinary stone disease include maximum stone clearance rate and less perioperative morbidity. The latest American Urological Association guidelines recommended percutaneous nephrolithotomy (PCNL) or extracorporeal shock wave lithotripsy (ESWL) for treatment of a pediatric renal stone exceeding 2 cm (6). However, ESWL has lost its popularity in treatment of large stones due to poor clearance rate (7, 8). Miniaturization of PCNL in recent years has diminished its perioperative comorbidities through decreasing the size of the access sheath tract (4, 9). Since there are few studies investigating this approach in the pediatric population, the aim of this study was to evaluate the postoperative outcomes of mini-PCNL (MPCNL) in terms of safety and efficacy in a single-referral center in Southern Iran.

## Methods

### Study Design

This study was approved by the ethics committees of Shiraz University of Medical Sciences (Approval code ofIR.sums.med.rec.1398.459) and performed in accordance with the Declaration of Helsinki. In this retrospective cross-sectional study, the pediatric patients who had undergone MPCNL between September 2012 and January 2020 in our referral centers (Nemazi Teaching Hospital and Ali-Asghar Teaching Hospital, Shiraz, Southern Iran) were considered for this study. During this period, 112 patients under age 18, who had failure of ESWL and/or their parents refused to do it, underwent MPCNL. Almost all patients were referred to our clinic by a pediatric nephrologist. They had preoperative evaluation of the urinary system including ultrasonography and plain abdominal and pelvic X-ray (KUB) or excretory urography for lucent stones and low-dose non-contrast CT scan with reconstruction limited to the kidneys (10). Also, complete blood count (CBC), renal function test (BUN and creatinine), and urine analysis and culture were done. Positive cultures were treated with appropriate antibiotic and admitted with sterile urine for operation. All patients were admitted 6 h before operation and received parenteral hydration and a single dose of prophylactic antibiotic.

#### Inclusion Criteria

The inclusion criteria were age under 18 years, normal renal function, renal stones more than 10 mm, and history of previous ESWL failure.

#### Exclusion Criteria

The exclusion criteria were patients with uncorrected coagulopathy, active urinary tract infection (UTI), patients who had undergone transplant or urinary diversion, and congenital abnormalities.

### Operation

The procedure was done under general anesthesia. In lithotomy or supine with abducted thigh position, a ureteral catheter 3 Fr or 4 Fr was inserted into the kidney and fixed to the urethral Foley catheter. Then, the patient was switched into prone position. After proper padding of the chest, abdomen, knee, and ankle, the patient was draped with sterile coverage. Then, diluted contrast was injected through a ureteral catheter, and under fluoroscopic guidance (C-arm fluoroscopy image intensifier), the pelvicalyceal system (PCS) was visualized. In patients whose color Doppler US guide with a 3.5-MHz probe (BK Medical) was used for puncture, saline was injected for better visualization of the PCS. A Chiba needle 18G was inserted in the target calyx, and a 0.035-inch J-tip guide wire was passed through the needle into the PCS and ureter, if possible. Tract dilation was performed using Alken telescopic dilators up to 18 Fr and Amplatz sheath 18 Fr (11). Nephroscopy was done with 15 Fr (Wolf) nephroscope. Lithotripsy was done with pneumatic lithoclast, and its particles were removed by forceps. Warm saline solution was used as irrigation for prevention of possible hyponatremia and hypothermia. Stone-free status was checked at the end of operation by fluoroscopy and US. Tubeless MPCNL was used in the patients with single-tract access, no significant residual stones, no significant perforation, minimal bleeding, and no requirement for a secondary procedure (12). Ureteral stent and urethral Foley catheter were removed 12 to 24 h postoperatively. Nephrostomy tubes were removed on the second day after operation. A plain abdominal film (KUB X-ray) or US was done on the day after operation, and residual stones, if presented, were followed for spontaneous passage of <4–5 mm stones and possible second-look nephroscopy (second-look nephroscopy was done 24 h after MPCNL, which was performed percutaneously through the previously established tract in an attempt to render the patient stone free).

### Data Collection

The patients' profiles were reviewed for data collection. Demographic data including age and sex were recorded. Preoperative information was previous history of stone or other surgery on the involved kidney, history of ESWL, solitary kidney, radiopacity of stone, hydronephrosis, stone laterality (left, right), stone location, and stone size. The mean stone size was assessed by preoperative low-dose non-enhanced computed tomography (CT) scan and determined by the largest diameter in the coronal view. Laboratory data consisted of blood urea nitrogen, creatinine, and hemoglobin that were measured before the surgery and 6 h after the operation. Operation and postoperative data included results and complications such as operation time, fluoroscopy screening time, stone-free status (KUB X-ray or ultrasound the day after the operation), hospitalization period, DJ insertion, ureteroscopy, redo-nephroscopy, fever, blood transfusion, infection, and other complications. Using the Modified Clavien grading system (13), postoperative complications were classified into fever as grade I, blood transfusion need, urine leakage, and urinary tract infection as grade II, double-J placement for urine leakage, ureteroscopy, and redo-PCNL as grade III, urosepsis and neighboring organ injury as grade IV, and death as grade V.

### Statistical Analysis

Continuous variables were presented using mean and standard deviation. Categorical variables were presented using numbers and percentages. All statistical analyses were done using SPSS software (IBM SPSS, version 13).

## Results

Of 112 patients, 69 were boys and 43 girls. Their mean age was 8.6 years (14 months to 18 years). Mean stone size was 20 mm (14–34 mm); 64 patients had left and 48 had right side stone. Seventy-four cases had renal pelvis stone, 22 had pelvis and lower pole, and 16 had staghorn. Thirty-six patients had a history of previous renal surgery (PCNL or open nephrolithotomy) on the same kidney, and 26 had a history of ESWL on the kidney. Ninety-eight had opaque, and the remaining 14 had lucent stone. Eight patients had solitary kidney. Seventy-seven of them had mild, 26 moderate, and 9 severe degrees of hydronephrosis on preoperative imaging studies (Table 1).

**Table 1:** Demographic characteristics of 112 pediatric patients who underwent mini-percutaneous nephrolithotomy (MPCNL).

Gender^a^	
Male	69 (61.60%)
Female	43 (38.39%)
Age (years)^b^	8.6 ± 6.1, 14 months to 18 years
Previous ESWL^a^	26 (23.21%)
Previous surgery on involved kidney^a^	36 (32.14%)
Solitary kidney^a^	8 (7.14%)
UTI^a^	17 (15.17%)
Stone size (mm)^b^	20.7 ± 8.5, 14–34
Stone location^a^	
Renal pelvis	74 (66.07%)
Lower pole	22 (19.64%)
Staghorn	16 (14.28%)
Stone laterality^a^	
Right	48 (42.85%)
Left	64 (57.14%)
Radiopacity of stone^a^	
Opaque	98 (87.50%)
Lucent	14 (12.50%)
Hydronephrosis^a^	
Mild	77 (68.75%)
Moderate	26 (23.21%)
Severe	9 (8.03%)
Preoperative HB (g/dl)^b^	13.4 ± 2.6, 11–15.6
Preoperative BUN (mg/dl)^b^	16 ± 3.2, 12–21
Preoperative creatinine (mg/dl)^b^	1.3 ± 0.36, 0.8–1.5

a *Data were presented as n (%)*.

b *Data were presented as mean ± SD, range*.

*ESWL, extracorporeal shock wave lithotripsy; HB, hemoglobin; mm, millimeter; UTI, urinary tract infection; BUN, blood urea nitrogen*.

The mean operation time was 65 min (35–100 min), and mean radiation was 0.6 min (0.2–1.4 min). Nephrostomy tube was inserted in 17 (15.17%) patients, tubeless MPCNL (having externalized ureteral catheter) in 70 patients including 6 patients who had double-J stent (62.50%), and totally tubeless in 25 (22.32%) cases.

Low-grade fever was detected in 14 patients (12.5%). Four patients needed packed cell transfusion, and two patients had increased level of creatinine, which improved with conservative management. One patient developed urosepsis that resolved with antibiotic therapy. None of the patients had kidney perforation or other organ injury or death (Table 2).

**Table 2:** Outcome and complications of MPCNL surgery of 112 pediatric patients.

Stone clearance status^a^	
Early stone free	101 (90.17%)
Final stone free	107 (95.53%)
Access method^a^	
Pure fluoroscopy	78 (69.64%)
Combined fluoroscopy/US	25 (22.32%)
Pure US	9 (8.03%)
Operation time (min)^b^	65 ± 28, 35–100
Fluoroscopy screening time (min)^b^	0.6 ± 0.46, 0.2–1.4
Postoperative nephrostomy tube status^a^	
Nephrostomy	17 (15.17%)
Tubeless:64/DJ:6	70 (62.50%)
Totally tubeless	25 (22.32%)
Postoperative hospitalization (h)^b^	36 ± 12, 24–56
Complications grade 1^a^	
Fever	14 (12.50%)
Grade 2^a^	
Blood transfusion	4 (3.57%)
Rise of blood creatinine	2 (1.78%)
Urine leakage	3 (2.67%)
Infection (UTI)	3 (2.67%)
Grade 3^a^	
DJ placement for urine leakage	2 (1.78%)
Residual	6 (5.35%)
Ureteroscopy	2 (1.78%)
Redo-MPCNL	3 (2.67%)
Grade 4^a^	
Urosepsis	1 (0.89%)
Visceral injury	0 (0.00%)
Grade 5^a^	
Death	0 (0.00%)

a *Data were presented as n (%)*.

b *Data were presented as mean ± SD, range*.

*DJ, double J; MPCNL, mini-percutaneous nephrolithotomy; UTI, urinary tract infection; US; ultrasound; wks., weeks*.

Early stone-free rate (SFR) after operation was 90.2% (101 patients) on the first day after operation in accordance with KUB X-ray or US (14), but 6 patients had residual fragment <5 mm, which passed spontaneously 2 weeks after the operation (total SFR reached 95.3% without any axillary procedure). Three patients underwent second-look nephroscopy, and ureteroscopy was done for two patients due to migrated stone fragments to the distal ureter.

## Discussion

The results of this study demonstrate high clearance rate and acceptable complication rate of MPCNL procedure in patients under 18 years of age. Nephrolithiasis in children is increasing, and treatments have progressed to minimal invasive modalities including ESWL and PCNL, with its advancement to super minimal invasive and laparoscopy, instead of open surgery such as in adults even for nephrectomy (15, 16). PCNL, as one of the minimal invasive ways for treatment of renal stones, has progressed and modified since 1976 (17). Renal access has changed from pure fluoroscopic to combined fluoroscopic/ultrasound and pure ultrasound guided to minimize the surgeon and patient radiation exposure (18, 19). The technology of miniaturization of the access sheath has progressed recently, and the miniaturized PCNL can currently be classified into mini-PCNL (≤22 Fr), Chinese mini-PCNL (14–20 Fr), super-mini-PCNL (10–14 Fr), ultra-mini-PCNL (11–13 Fr), micro-PCNL (4.8 Fr), and mini-micro-PCNL (8 Fr) (20). Jackman was the first to perform MPCNL for adult patients in 1997 (21).

Complications of PCNL include bleeding, infectious complications, rupture of the PCS, urinary system leakage, thoracic complications, other organ injury (colon, bowel, spleen, liver), and postoperative pain (22, 23). It is established that MPCNL has the same effectiveness and less complications compared with standard PCNL (17, 24). SFR does not significantly differ between these two procedures. Bleeding, blood transfusion need, and postoperative hospitalization are less in MPCNL (17). Operation time is slightly more in MPCNL. Fever, postoperative pain, kidney, and another organ injury are less in MPCNL (24).

Wah et al. (25) performed MPCNL on 23 pediatric patients. The median stone burden was 3.44 cm^2^, 10 on the right and 13 on the left. Their primary SFR was 83.6%, which increased to 90.5% after treating the residual fragments. One of their patients developed postoperative hydrothorax and two of them had urinary tract infection. We had early SFR about 90% and total SFR 95.3%, which was higher than other studies, and 5.3% residual stone fragment. Farouk et al. (26) performed a prospective study on 108 pediatric patients with 1- to 2-cm renal stone and randomized them into two groups to compare MPCNL and ESWL. Those who underwent MPCNL had 88.9% SFR after the first session surgery and reached 92.59% SFR after the second-look PCNL. However, the ESWL group had 55.6% after the first session and reached 88.89% SFR after the third session of ESWL (26). Another study compared the outcomes of super-MPCNL and retrograde intra-renal surgery (RIRS) for children with upper urinary tract calculus. Those who underwent RIRS had a significantly longer operating time (76.3 vs. 53.9 min) and postoperative hospital stay (4.2 vs. 2.9 days), less SFR (60.0 vs. 94.4%), and more re-treatment rate (20.0 vs. 0.0%) (27).

Another retrospective study was done on 163 patients who underwent MPCNL. They reported 95.7% SFR, 37-min mean operation time, 18.4-mm mean stone size, and 14.6% postoperative fever (28). In our study, 12.5% of the patients developed fever after surgery, and one of them had urosepsis. Sofimajidpour et al. (29) reported the outcome of 22 pediatric patients who underwent ultra-mini-PCNL. SFR was 95.5% among their patients, 18.2% had postoperative fever, and none of them developed septicemia. Liu et al. had a study to explore the risk factors of sepsis following MPCNL. Twenty out of 834 studied patients had developed septic shock. Of them, three patients expired due to multiple organ dysfunction syndromes. They found that postoperative septic shock was associated with female sex (OR = 1.055, *P* < 0.001) and diabetes mellitus (OR = 4.192, *P* = 0.001) (30). All of our patients were cured with antibiotic therapy and conservative managements. Children may develop hyponatremia and hypothermia; it is recommended to use warm saline for irrigation. Although, some studies showed that distilled water was safe in adult PCNL, saline is the standard solution (31). None of our patients had developed these problems in this study.

The current study had several limitations. First, this is a retrospective observational study, which needs validation with larger sample size, but in prospective studies. Second, the natural history of residual fragments in children is not well-known and is estimated the same as adult urolithiasis, which may not be similar for pediatric cases. Third, our study had a few subjects <5 years of age. Fourth, we did not use the multivariable model (The multivariable models were studied in previous articles. Additionally, it could help avoid crowdedness and make this study easier to read and comprehend since we aimed to evaluate the MPCNL in pediatric patients). Next, there was no access to data on stone composition (The use of infrared spectrophotometry had not been routinely used by the laboratory in our center, and most of the stone analyses were carried out in a private laboratory clinic, and we do not have access to these data); finally, there was no control group for comparison of MPCNL with other treatment modalities.

## Conclusion

The results of our study reveal that MPCNL in children is a relative safe procedure with acceptable complication and appropriate surgery outcomes.

## Data Availability Statement

The raw data supporting the conclusions of this article will be made available by the authors, without undue reservation.

## Ethics Statement

The studies involving human participants were reviewed and approved by the ethics committees of Shiraz University of Medical Sciences (Approval code# IR.sums.med.rec.1398.459). Written informed consent to participate in this study was provided by the participants' legal guardian/next of kin.

## Author Contributions

MH: investigation, resources, writing-reviewing and editing, and supervision. DI, AAl, and AE: investigation and resources. AH: methodology, software, and formal analysis. MB: software and data curation. AAd and FA: conceptualization, writing-original draft preparation, software, and data curation. All authors contributed to the article and approved the submitted version.

## Conflict of Interest

The authors declare that the research was conducted in the absence of any commercial or financial relationships that could be construed as a potential conflict of interest.
